# Quality Assessment of Digital Health Apps: Umbrella Review

**DOI:** 10.2196/58616

**Published:** 2024-10-10

**Authors:** Maciej Marek Zych, Raymond Bond, Maurice Mulvenna, Jorge Martinez Carracedo, Lu Bai, Simon Leigh

**Affiliations:** 1 School of Computing Ulster University Belfast United Kingdom; 2 School of Electronics, Electrical Engineering and Computer Science Queen's University Belfast Belfast United Kingdom; 3 ORCHA Daresbury United Kingdom

**Keywords:** mHealth assessment, digital health, quality assessment, health apps quality, assessment criteria, evaluation criteria, health apps criteria, assessment, digital health app, app, umbrella review, risk, mobile phone, frameworks

## Abstract

**Background:**

With an increasing number of digital health apps available in app stores, it is important to assess these technologies reliably regarding their quality. This is done to mitigate the risks associated with their use. There are many different guidelines, methods, and metrics available to assess digital health apps with regard to their quality.

**Objective:**

This study aimed to give a holistic summary of the current methods and “condition agnostic” frameworks that are broadly applicable for the quality assessment of all digital health apps.

**Methods:**

A systematic search of literature was conducted on 4 databases: Scopus, PubMed, ACM Digital Library, and IEEE Xplore. We followed the PICOS (Population, Patient, or Problem; Intervention; Comparison; Outcomes; and Study Design) and PRISMA (Preferred Reporting Items for Systematic Reviews and Meta-Analyses) methodologies when conducting this umbrella review. The search was conducted on January 26, 2024, for review articles published between 2018 and 2023. We identified 4781 candidate papers for inclusion; after title and abstract screening, 39 remained. After full-text analysis, we included 15 review articles in the full review.

**Results:**

Of the 15 review articles, scoping reviews were the most common (n=6, 40%), followed by systematic reviews (n=4, 27%), narrative reviews (n=4, 27%), and a rapid review (n=1, 7%). A total of 4 (27%) review articles proposed assessment criteria for digital health apps. “Data privacy and/or security” was the most mentioned criterion (n=13, 87%) and “Cost” was the least mentioned criterion (n=1, 7%) for the assessment of digital health apps. The Mobile App Rating Scale was the most frequently used framework for quality assessment of digital health apps.

**Conclusions:**

There is a lack of unity or consolidation across identified frameworks, as most do not meet all the identified criteria from the reviewed articles. Safety concerns associated with the use of digital health apps may be mitigated with the use of quality frameworks.

## Introduction

Digital health apps have a potential to make health care more accessible to people of different age groups living with a wide range of health-related conditions. Currently, there are ongoing efforts by the National Health Service (NHS) in the United Kingdom to enhance the use of digital health technologies. These efforts were outlined in the NHS long-term plan [1]. Moreover, these efforts were accelerated by the COVID-19 pandemic [2]. There are approximately 250 new digital health apps available in app stores per day, with a total of approximately 350,000 digital health apps on the market as of 2021 [3]. As digital health apps rise in popularity, so do the risks associated with their use. Digital health apps that are classified as “medical devices” [4] in the United Kingdom are strictly regulated, but digital health apps that are classified as “wellness apps” do not face such regulations.

There are organizations that produce guidelines and frameworks regarding the development and assessment of digital health apps. These include the International Organization for Standardization (ISO): Health and Wellness Apps—Quality and Reliability [5] and the National Institute for Health and Care Excellence (NICE): Evidence Standards Framework [6]. Digital Technology Assessment Criteria (DTAC) [7] is an NHS-developed framework that is being used to provide criteria for the assessment of digital health apps. However, some of the guidelines may be open to interpretation, and there are frameworks that are currently being used to assess digital health apps for their quality.

A scoping review published in 2023 [8] examined the problems and barriers related to the use of digital health apps. It found that “validity,” “usability,” “technology,” “data privacy and security,” and “individuality” were addressed in several studies and are partly considered in quality assessment. “Use and adherence,” “patient-physician relationship,” “knowledge and skills,” “implementation,” and “costs” of digital health apps were rarely extensively studied. Furthermore, a systematic review published in 2021 [9] found security challenges when developing digital health apps. These and other quality problems surrounding digital health apps may be mitigated by rigorously assessing their quality.

Digital health apps are complex, and the usual methods for assessing medicines (survival, quality of life, and cost), as recommended by NICE, may not be sufficient to monitor the broad spectrum of potential issues arising from their use. Therefore, more specific requirements for assessment of quality are required. However, there is a lack of consensus on how best to achieve this.

In this umbrella review, we define quality as “compliance with best practice standards.” The objective of this umbrella review was to give a holistic summary of the current methods and “condition agnostic” frameworks that are broadly applicable for the quality assessment of all digital health apps. Because several review articles have been published regarding the quality assessment of digital health apps, or aspects related to this, we conducted an umbrella review to provide a holistic view of how digital health apps are currently being assessed and where they can improve. This review can be informative to digital health researchers and assessment framework developers. In this umbrella review, we included systematic reviews, scoping reviews, rapid reviews, and narrative reviews.

## Methods

### Search Strategy and Criteria

For the systematic search of literature, the PICOS (Population, Patient, or Problem; Intervention; Comparison; Outcomes; and Study Design; see Textbox 1) and PRISMA (Preferred Reporting Items for Systematic Reviews and Meta-Analyses) methodologies have been used. The following search has been conducted using the Scopus, PubMed, ACM Digital Library, and IEEE Xplore databases with the following search objective: find literature, systematic, or scoping review articles for quality assessment of digital health apps. The search for review articles was conducted on January 26, 2024.

**PICOS (Population, Patient, or Problem; Intervention; Comparison; Outcomes; and Study Design) methodology for systematic search of previous or related literature reviews.**

**Inclusion criteria**

- Problem: Review of quality assessment tools for digital health apps, full article, in English, from 2018 to 2023.
- Intervention: Quality assessment frameworks or criteria review applicable to all digital health apps.
- Comparator: Not applicable (N/A)
- Outcome: Review articles regarding quality (or aspects of quality, eg, usability) assessment frameworks or criteria applicable to all digital health apps.
- Study design: Search databases included Scopus, PubMed, ACM Digital Library, and IEEE Xplore. Search query for article title and abstract: ( ( mhealth OR ehealth OR m-health OR e-health OR “mobile health” OR “electronic health” OR “health app*” OR “medical health app*” OR “digital health app*” OR “digital health product*” OR “digital health intervention*” OR “digital health technolog*” OR “digital health solution*” ) AND ( assurance* OR assessment* OR evaluation* OR audit* OR framework* ) AND ( review* OR assessment* ) )

**Exclusion criteria**

- Problem: Not quality assessment of digital health apps, conference paper, book or book chapter. Not in English.
- Intervention: Not a quality assessment frameworks or criteria review.
- Comparator: N/A
- Outcome: No information on quality assessment frameworks or criteria. Does not focus on digital health apps. Articles targeting specific users (eg, women or adolescence). Focuses on specific feature or category (condition area). Frameworks that focus on user acceptance of technology.
- Study design: N/A

Multimedia Appendix 1 presents the PRIOR checklist and Multimedia Appendix 2 presents the exact search queries that were used for each of the databases.

### Study Screen

This study used PICOS methodology to set the inclusion and exclusion criteria (Textbox 1). The review articles included in this study were initially screened by title and abstract. If the review article focused on quality (or its aspect) assessment of digital health apps, it was read in full. If the article met inclusion criteria and none of the exclusion criteria (Textbox 1), it was included in the study. This systematic search followed PRISMA guidelines when screening the articles, where a step-by-step process was set forth. The Rayyan tool has been used to remove article duplicates.

### Critical Appraisal

Because 4 (27%) of the 15 review articles were systematic reviews, for those reviews, the Joanna Briggs Institute (JBI) critical appraisal of systematic reviews [10] has been used (see Multimedia Appendix 3). This has been done to assess the quality of the systematic reviews to determine whether they should be included in this umbrella review.

### Data Extraction

The characteristics of this study follow the applicable protocols for the umbrella review from the *JBI Manual for Evidence Synthesis* [11]. The following information has been extracted: author-year, objectives, total sample size, number of sources searched, date (year) range of searched and included studies, number of studies included, methods of analysis, and key findings (see Multimedia Appendix 4).

### Data Synthesis

In this study, we did not synthesize results statistically. Instead, we narratively synthesized key findings of each of the reviews. This was done due to the presumed heterogeneity of the included reviews.

## Results

### Overview

**Figure 1:**
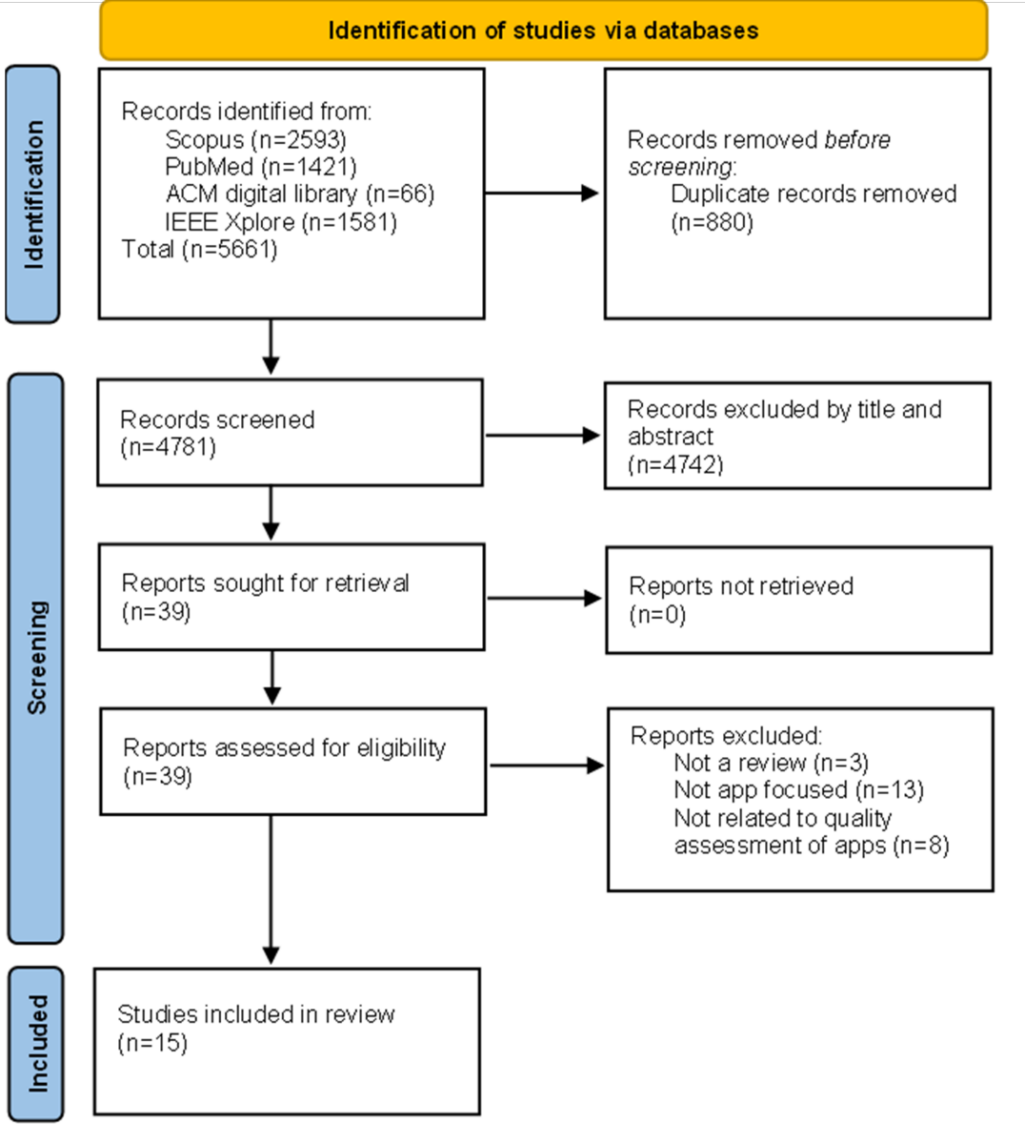
PRISMA (Preferred Reporting Items for Systematic Reviews and Meta-Analyses) diagram of reviews selected for inclusion in the umbrella review.

Figure 1 shows the PRISMA process of selecting article reviews to be included in the study. The search queries used for each of the databases searched (Scopus, PubMed, ACM Digital Library, and IEEE Xplore) are available in Multimedia Appendix 2. After duplicates were removed, the articles were reviewed by title and abstract, and were included only if they were related to quality assessment of digital health apps and did not meet the exclusion criteria outlined in Textbox 1. Afterward, 39 review articles were read in full and 15 met the inclusion criteria and were included in this umbrella review. This review was not registered and a protocol for systematic reviews was not prepared.

Mendeley (Elsevier) was used to manage all the included review articles (n=15) in the study. The 15 review articles were published between 2018 and 2023. Scoping reviews were the most common (n=6, 40%), followed by systematic reviews (n=4, 27%), narrative reviews (n=4, 27%), and a rapid review (n=1, 7%). Multimedia Appendix 5 depicts which criteria the review articles focused on.

### The Assessment Criteria

The objective of this umbrella review was to give a holistic summary of current methods and frameworks used for quality assessment of digital health apps. Table 1 depicts 4 (27%) of the 15 review articles that reported on assessment criteria for digital health apps. Of the 4 review articles, 2 (50%) outlined what was being assessed with frameworks [12,13] and 2 (50%) focused on what should be assessed [14,15]. The remaining 11 review articles included in this umbrella review either focused on a specific criterion (eg, usability), the methods of assessment (eg, Likert scale), or referenced the criteria of a review article already accounted for (Woulfe et al [16] references the Nouri et al [13] criteria). Nouri et al [13] stated that different articles defined assessment criteria differently. For example, usability was mentioned in 14 articles that were reviewed. One article considered usability a subclass of ease of use; other articles placed ease of use under usability.

**Table 1:** Framework criteria used for the assessment of digital health apps.

Authors, Year	Obtained by^a^	Criteria (assessment domains)
Hensher et al, 2021 [12]	Assessing frameworks	Clarity of purpose of the app Developer credibility Content or information validity User experience User engagement or adherence and social support interoperability Value Technical features and support Privacy, security, ethical, or legal Accessibility
Nouri et al, 2018 [13]	Assessing frameworks	Design Information or content Usability Functionality Ethical issues Privacy and security User-perceived value
Moshi et al, 2018 [14]	Preset or developed	Six core criteria: Current use of the technology Description and technical characteristics Effectiveness Safety Cost and cost-effectiveness Organizational aspects Three optional criteria: Legal aspects ethical aspects social aspects
Lagan et al, 2021 [15]	Preset or developed	Mobile health Index and Navigation Database (MIND) criteria: App origin App functionality Inputs and outputs Privacy and security Evidence and clinical foundation Features and engagement style App use Interoperability and data sharing

^a^“Obtained by” states whether assessment criteria have been identified by assessing assessment frameworks or preset or developed by other means.

Hensher et al [12] and Nouri et al [13] identified criteria by assessing assessment frameworks. Moshi et al [14] and Lagan et al [15] had their own set of criteria and compared assessment frameworks against those criteria. Moshi et al [14] developed a checklist for health technology assessment for mobile medical apps based on previous research [17,18]. Lagan et al [15] used the previously proposed Mobile health Index and Navigation Database (MIND) [19], which was developed with inputs from clinicians, patients, family members, researchers, and policy makers with the aim of providing clinically relevant criteria. Nouri et al [13] stated that there may never be complete digital health apps assessment criteria because these criteria apply to digital health apps that are changing in development continuously.

### Data Privacy or Security

Thirteen (87%) of the 15 review articles [12-16,20-27] mention the data privacy or security of digital health apps. Three reviews [13,20,21] found that reviewed articles often considered privacy and security together when evaluating digital health apps. Nurgalieva et al [21] highlighted that although privacy and security overlap, security relates to protection against unauthorized access to data, and privacy is an individual’s right to maintain control of their personal data. The review points out that exclusively focusing on security can lead to increased surveillance, which can introduce privacy risks. Furthermore, Nouri et al [13] stated that interpretation of privacy, security, and safety considerations differed when examining different quality assessment tools or methods.

Nurgalieva et al [21] found that methods used to evaluate security are more technical, whereas methods used to evaluate privacy are more user oriented. Some of the review articles [15,21] pointed out that there appears to be greater effort to assess privacy. However, Muro-Culebras et al [22] found lack of assessment regarding developer transparency and policies regarding user data privacy and security. Lagan et al [15] found that most of the examined assessment frameworks (43/79, 54%) included privacy-related questions. Hensher et al [12] also found privacy and security to be frequently addressed in assessment frameworks.

Grundy [23] discussed how guidelines such as General Data Protection Regulation (GDPR) [28] rely on the users’ knowledge and “notice and consent” model. Grundy [23] referenced a study published in 2020 [29] that discusses whether GDPR is fit for purpose, as it assumes that users can know why and how their data are being collected and shared. GDPR also assumes that individual app users can control how their personal data are being processed. Grundy [23] stated that the majority of digital health apps fail to provide assurances around privacy and security. On the other hand, Carmi et al [25] focused on the interpretation of GDPR for mobile health.

Galvin and DeMuro [26] discussed how recent literature has shown that aggregated data that were previously considered deidentified have been shown to be reidentifiable. The review states that data storage and transmission of mobile health data remain a security concern. Moreover, the review points out that because of the lack of privacy policy, or because of the complex language used in such policies, there may be lack of consumer informed consent when using mobile health apps.

### Clinical Assurance, Credibility of Information, or Evidence

Eleven (73%) of the 15 review articles [12-16,22-24,26,27,30] included in this umbrella review mentioned clinical assurance, credibility of information of digital health apps, and evidence. Hensher et al [12] stated that in 2 studies included in their review, third-party sponsorships were deemed important due to a possible conflict of interest between developers of an app and sponsors, affecting developers’ credibility. Moshi et al [14] found that credibility of information of mobile medical apps was assessed by 15 (33%) of 45 frameworks. Moreover, 27 (60%) of 45 frameworks included questions about mobile medical apps’ source of information. None of the frameworks assessed the health impact of mobile medical apps that provide diagnostics or information. The review concluded that none of the included frameworks met all the health technology assessment criteria (see Table 1—Moshi et al [14]) set forth. A total of 3 (7%) of 45 frameworks specifically asked about randomized controlled trials. Lagan et al [15], which expands on the work done by Moshi et al [14], has shown that more frameworks include questions around clinical foundation than in their previous article published in 2019 [31], indicating an increase of interest around clinical foundation assessment.

Muro-Culebras et al [22] stated that many authors use their own personalized questionnaires for assessment, specifically designed to assess the characteristics of their specific digital health apps. The review article stated that because the approach is a personalized questionnaire, there is greater flexibility than in other generic tools. However, lack of validation and reliability (such as inter-rater reliability) raises questions about their suitability for digital health app assessment. Muro-Culebras et al [22] concluded that highly validated tools for the assessment of digital health apps are still a largely unexplored topic in the market. Similarly, Grundy [23] stated that there is a lack of measurement tool validation. Furthermore, users do not appear to have much awareness of the source or validity of health information of a digital health app. Akbar et al [24] found that digital health apps lack domain expert involvement regarding the app content and provide poor evidence base and poor validation.

### User Experience, Value, Efficacy or Effectiveness, or Engagement

Eleven (73%) of 15 review articles [12-16,22-24,27,30,32] mentioned user experience, value, efficacy, or engagement. Hensher et al [12] found that aspects of user experience have been frequently assessed by the assessment frameworks. Moreover, the review stated that there is limited evidence on how to evaluate the value domain in the literature. They speculate that this could be due to the current landscape of digital health apps’ market being fast and evolving, and the subjectivity of value. The review also stated that studies to demonstrate apps’ efficacy and value for money are not undertaken often. Moshi et al [14] stated that of 45 frameworks included in the review, all assessed effectiveness to some extent. Of 45 frameworks, 11 assessed user satisfaction and 30 assessed technical efficacy of mobile medical apps.

Maramba et al [32] focused on methods of usability testing of eHealth applications. Questionnaires, task completion, think-aloud protocol, interviews, heuristic testing, and focus groups were the most frequently used methods of assessment, whereas methods such as eye tracking were rarely used. The review concludes that more investigation needs to be made into assessing usability of eHealth applications. Muro-Culebras et al [22] found that usability among the 8 frameworks assessed in the review was commonly assessed together with engagement, aesthetics, or functionality. Moreover, the review found that 2 (75%) of the 8 frameworks had a user assessor and a professional assessor version. Woulfe et al [16] point out that many digital health apps are not based on any behavior change theory, and in many cases, effectiveness is inadequately assessed. They also address the possibility of using different assessment methodologies in high-, low-, and middle-income countries.

Lagan et al [15] point out that subjective user experience may limit generalizability and standardization of frameworks. This is because assessment would reflect the experience of the assessor. The review further points out that although subjective in nature, information on user friendliness, visual appeal, and interface design may be of great interest to the user and a good predictor of user engagement.

Akbar et al [24] stated that users should be involved in usability testing of digital health apps. Their review indicates that consumers were able to identify many critical issues with digital health apps, such as incorrect information, inappropriate response to their needs, gaps in features, and faults with alarms. Akbar et al [24] suggest that allowing users who are consumers of digital health apps to be involved in usability testing will enable the identification and resolution of usability problems before the apps are made available to the public.

Grundy [23] pointed out that the assessment frameworks mainly focused on content quality and usability, with less attention given to design, security and privacy, functionality, user-perceived value, and ethical issues. Nouri et al [13] stated that usability was treated in different ways by different articles. For example, one article considered usability a subclass of ease of use, and other articles placed ease of use under usability. Azad-Khaneghah et al [27] found that many of the usability and quality rating scales are targeted at professionals. Moreover, the review found that System Usability Scale (SUS) was the most widely used framework or scale (12/40 studies), mainly due to its simplicity. Similarly, Hajesmaeel-Gohari et al [30] found that general questionnaires with fewer questions and higher reliability, such as SUS, have been used more often. Furthermore, the review recommends using frameworks such as the mHealth App Usability Questionnaire [33] that were specifically designed for mobile apps, unlike the SUS.

### Safety

Safety (in different contexts) has been mentioned by 9 (60%) of the 15 review articles [12-14,16,20-24]. Safety and risks of digital health apps can arise from different factors. Akbar et al [24] elaborate on different safety concerns and risks associated with the use of digital health apps. The review found 67 safety concerns related to the quality of content, which are grouped into the following 5 categories: incorrect information, incomplete information, variation in content, incorrect output, and inappropriate response to consumer needs. Akbar et al [24] found 13 safety concerns related to software functionality. These are grouped into 5 more categories: gaps in features, lack of validation for user input, delayed processing, response to health dangers, and faulty alarms. Akbar et al [24] further discuss consequence of safety concerns, for example, how one of the digital health apps led to dangerous levels of alcohol consumption among a group of 341 students. Overall, many of the frameworks do not cover the necessary criteria to quality assess digital health apps [14,15]. Moshi et al [14] consider safety as part of the assessment criteria for digital health apps (see Table 1).

### Features or Functionality

Six (40%) of the 15 review articles [12-16,27] considered feature or functionality as criteria to be assessed, referred to as technical characteristics by Moshi et al [14]. Furthermore, Nurgalieva et al [21] mention that feature assessment was common when assessing security and privacy. Lagan et al [15] stated that features related to ease of use and visual appeal may be the most important drive of user engagement of mental health apps. Moreover, “subjective questions” around user friendliness, visual appeal and interface design, although difficult to standardize and to assess, may be the greatest predictor of user engagement. Lagan et al [15] and Hensher et al [12] identified digital health apps’ features as part of the assessment criteria (Table 1). Moshi et al [14] and Nouri et al [13] included functionality and technical characteristics, respectively, as part of their assessment criteria for digital health apps. The terms “features,” “functionality,” and “technical characteristics” seem to overlap in the review articles.

Nouri et al [13] stated that 2 (9%) of 23 review articles provided dynamic assessment criteria based on the use cases and features of specific digital health apps, meaning that the assessment criteria were selected for apps based on the use case. They provide an example of the criterion “accuracy of the calculations,” being used only when an app provides at least 1 calculation. Akbar et al [24] stated that many digital health apps had gaps in features that inadequately supported user tasks. They gave an example of “Tele dermatology” apps that did not account for allergies or current medication status.

### Cost

Cost as a criterion has been mentioned by 1 (7%) of the 15 review articles [14]. Moshi et al [14] stated that there may be a cost barrier to accessing mobile medical apps because they may contain in-app purchases or require a subscription. They further stated that only 1 (2%) of 45 frameworks assessed cost-effectiveness (in terms of economic assessment) and 11 (24%) of 45 reviewed the cost in terms of price to download or in-app purchases. Nine (60%) of the 15 review articles [12,13,16,20,21,23,24,26,32] mentioned the cost of digital health apps, the cost of data breaches, or equipment cost, but not as a criterion for quality assessment.

### Ethical or Legal Issues

Ethical or legal concepts have been mentioned by 10 (67%) of the 15 review articles [12-14,16,20,21,23-26]. Nurgalieva et al [21] and Benjumea et al [20] discuss ethical and legal concepts regarding data and data breaches. Nurgalieva et al [21] discuss how in their review of privacy and security of digital health apps, there was a considerable lack of discussion in the reviewed articles (n=83) regarding privacy or security ethics. Grundy [23] stated that quality assessment frameworks give less attention to ethical issues. The author also stated that legal compliance around content and intellectual property is an aspect of quality regarding commercial apps.

Moshi et al [14] discuss health technology assessment criteria for the assessment of mobile medical apps, including ethical and legal aspects. The review found that 4 (9%) of 45 frameworks discussed legal aspects and 24 (53%) of 45 frameworks discussed ethics. Hensher et al [12] also included ethics and legal aspects as part of their assessment criteria (see Table 1). For example, in the reviews of both Moshi et al [14] and Hensher et al [12], ethical aspects included privacy policies and legal aspects with mention of disclaimers. Nouri et al [13] included ethical issues as part of the assessment criteria (see Table 1). Akbar et al [24] stated that health care professionals may hesitate to promote digital health apps in part due to legal issues.

### Assessment Methods and Metrics

Hensher et al [12] from Deakin University conducted a scoping review focusing on the time frame from 2011 to April 2020 using search terms that were synonyms of “health apps,” “evaluation,” and “frameworks” [12]. This review examined 97 evaluation frameworks and studies that included general digital health app evaluation frameworks, such as the Mobile App Rating Scale (MARS), and more domain-specific frameworks, such as the SUS and Software Usability Measurement Inventory (SUMI).

The scoring and rating techniques varied within the different frameworks, that is, 23% of frameworks used a 5-point scale, 6% a 3-point scale, 3% a 7-point scale, 2% a 4-point scale, and 1% a 10-point scale. A total of 24% frameworks did not elaborate on the scaling system, 20% used a mixed approach, 13% were dichotomous, and 8% did not use a scaling system [12]. Moreover, the frameworks’ scoring modalities also varied as 37% did not report a score, 23% used a mean score, 13% used a total sum, 11% used mixed approaches, 9% other, and 6% did not use scoring at all [12]. In Nurgalieva et al [21], it appears that the evaluation of self-declared data from app developers was the most common privacy assessment method.

Hensher et al [12] examined the domain or criteria needed to evaluate digital health apps and found that user experience together with information validity has been the most evaluated criteria. However, this scoping review included frameworks such as SUS and SUMI, and such frameworks that are designed to evaluate usability are not tailored to digital health apps. In their “count,” if a framework evaluated an aspect of user experience (UX), it was considered a scale for evaluating UX. Usability is only one aspect of UX.

Lagan et al [15] found that most evaluation frameworks for health apps were concerned with evidence, clinical foundation, and privacy. This study suggests that it is unclear whether engagement has been adequately predicted with the existing frameworks. The study also suggests that a balance between objective and subjective questions is a challenge for evaluation frameworks.

## Discussion

### Principal Findings

This umbrella review included 15 review articles that were obtained via a systematic search of the literature (see Textbox 1 and Figure 1). The objective of this review was to give a holistic summary of current methods and frameworks used for quality assessment of digital health apps. It appears that frameworks are a common way of assessing digital health apps. Four (27%) of the 15 review articles tried to establish appropriate assessment criteria for digital health apps. Two (50%) of these 4 review articles [12,13] reviewed frameworks for digital health app assessment and derived their criteria. The other 2 (50%) of these 4 review articles [14,15] had predefined criteria based on previous research in the area (see Table 1). In the review articles, there was a lack of discussion regarding the unethical use of dark patterns, defined by UX dictionary [34] as “deceptive design patterns used to mislead users to make them do something they would not do on their own. They are primarily used to generate sales, increase subscriptions, and hit target business numbers.” Also, there was no mention of equality, diversity, and/or inclusion.

MARS has been the most frequently used framework for quality assessment according to 2 review articles [12,27]. Across the review articles included in this study, 13 (87%) of 15 included data privacy or security as a criterion. Hence, data privacy or security was the most common criterion. The least mentioned criterion was cost, with 1 (7%) of 15 review articles mentioning it (see Multimedia Appendix 5). Having an overarching assessment framework would reduce the need to apply several separate frameworks in the pursuit of identifying digital health app quality. However, when using frameworks for the quality assessment of digital health apps, it is important to remember the various limitations of frameworks. For example:

1. A framework can contain a lot of questions that sometimes need to be answered in a specific order. Hence, using it may require training.
2. A framework may not “capture” all necessary aspects of a product or system for evaluation.
3. A framework may be better at “capturing” one aspect of a product or system, for example, effectively evaluate ease of use, but lack in clinical assurance.
4. Evaluation is never perfect; frameworks rely on good interrater and intrarater reliability.
5. Frameworks are limited by their domain or condition area, meaning a framework may be suitable to assess all digital health apps (such as Enlight [34]), but not pick up on a condition area that is specific to quality issues, such as insulin intake for diabetes apps.

Speculation can be made that health condition–specific frameworks may provide a more accurate view of quality. This is because specific features may be required by digital health apps targeted at a specific health condition. A framework for a specific health condition, for example, may include questions such as insulin intake for diabetic users of an app. A generic, all-encompassing frameworks may include questions such as “did the app development involve a relevant health expert?” which could only indicate, not verify, whether necessary features are included.

Woulfe et al [16] point out that a framework coined Enlight [34] is a far-reaching and all-encompassing framework for the assessment of digital health apps. However, Enlight includes many questions, more than other generic frameworks used for the assessment of digital health apps. Hence, using it may take more time and curtail its use. However, the use of such frameworks may help in the mitigation of variety of problems with digital health apps [8],9], whereas using inadequate assessment frameworks may lead to overlooking flaws associated with the use of digital health apps. Hence, choosing a framework that adheres to all or most of the criteria mentioned in Table 1, such as DTAC [7] or Enlight [34], should enable a selection of a good quality app, despite not being focused on a specific condition area.

When developing new quality assessment frameworks, it may be helpful for the assessor to assess related criteria together; however, on the flip side, it can create confusion. For example, Nurgalieva et al [20] pointed out that exclusively focusing on security can lead to increased surveillance, which can introduce privacy risks. It can be speculated that if one criterion is being assessed, such as security, then privacy is also being assessed, leading to the omission of questions or areas in the assessment where security and privacy may be in conflict. Hence, caution needs to be taken when merging criteria into one in a framework. Textbox 2 provides a list of recommendations to improve the development of quality assessment frameworks.

**Recommendations to improve the development of quality assessment frameworks.**

1. Standardize definitions of the criteria used for the quality assessment of digital health apps.
2. When choosing any framework, ensure that it has been validated regarding its content and inter-rater reliability.
3. Choosing a framework that adheres to all or most of the criteria mentioned in Table 1 should enable a selection of a good quality app.
4. Ensure that improving compliance with one criterion does not sabotage the other. For example, an increase in security does not sabotage data privacy.
5. Ensure that when frameworks combine multiple criteria into one, for example, data privacy and security, there are no omissions of questions related to the original criteria.
6. More focus on the unethical use of dark patterns in app design.
7. Include criteria related to equality, diversity, and inclusion in order to ensure that digital health apps are widely available to different groups of people.
8. Choosing a framework for a specific health condition may allow for the assessment of specific or necessary features that would not be covered by a generic framework.
9. Ensure that third-party sponsorships do not lead to conflict of interest between developers of an app and sponsors, thus affecting the developers’ credibility.
10. Ensure that the language in the privacy policy is easily understandable. Frameworks should point out when privacy policy of an app contains language that is unnecessarily unclear or vague.
11. Specifying the context in which safety concerns are being assessed can reduce confusion, for example, safety related to data privacy or evidence for clinical assurance.

### Limitations

This review is based on 15 review articles, most commonly scoping reviews (n=6, 40%), followed by systematic reviews (n=4, 27%), narrative reviews (n=4, 27%), and a rapid review (n=1, 7%). Using different search queries and searching wider publication dates could yield more results. The screening of articles was conducted by 1 coauthor. This review only included article reviews that contained the word “review” in the title. Any review about quality of digital health apps that was condition specific (eg, diabetes) was excluded from this umbrella review.

### Conclusions

The majority of frameworks do not meet all the criteria identified from the reviewed articles. Safety concerns associated with the use of digital health apps may be mitigated with the use of quality frameworks. Some criteria for the assessment of digital health apps may conflict with each other. For example, overly focusing on security may lead to privacy concerns. Research indicates that subjective questions, although difficult to standardized, may be the most useful when assessing engagement.

## Appendix

Multimedia Appendix 1 Preferred Reporting Items for Overviews of Reviews (PRIOR) checklist.

Multimedia Appendix 2 Search queries.

Multimedia Appendix 3 JBI critical appraisal of systematic reviews.

Multimedia Appendix 4 Article characteristics.

Multimedia Appendix 5 Criteria addressed by each paper.

## Abbreviations

DTAC: Digital Technology Assessment Criteria
GDPR: General Data Protection Regulation
ISO: International Organization for Standardization
JBI: Joanna Briggs Institute
MARS: Mobile App Rating Scale
MIND: Mobile health Index and Navigation Database
NHS: National Health Service
NICE: National Institute for Health and Care Excellence
PICOS: Population, Patient, or Problem; Intervention; Comparison; Outcomes; and Study Design
PRISMA: Preferred Reporting Items for Systematic Reviews and Meta-Analyses
SUMI: Software Usability Measurement Inventory
SUS: System Usability Scale
UX: user experience
WHO: World Health Organization

## References

[1] The NHS long term plan.

[2] The NHS long term plan and COVID-19. The Health Foundation.

[3] Kern J, Skye A, Krupnick M, Pawley S, Pedersen A, Preciado K Written consent of IQVIA and the IQVIA Institute. Digital Health Trends.

[4] Regulating medical devices in the UK. GOV.UK.

[5] ISO/TS 82304-2:2021 Health software Part 2: Health and wellness apps—quality and reliability. ISO.

[6] Evidence standards framework for digital health technologies. NICE.

[7] Digital Technology Assessment Criteria (DTAC). NHS.

[8] Giebel GD, Speckemeier C, Abels C, Plescher F, Börchers K, Wasem J, Blase N, Neusser S (2023). Problems and barriers related to the use of digital health applications: scoping review. J Med Internet Res.

[9] Aljedaani B, Babar MA (2021). Challenges with developing secure mobile health applications: systematic review. JMIR Mhealth Uhealth.

[10] (2017). Checklist for systematic reviews and research syntheses. JBI.

[11] Aromataris EC, Fernandez R, Godfrey C, Holly C, Khalil H, Tungpunkom P, Aromataris E, Lockwood C, Porritt K, Pilla B, Jordan Z (2024). Umbrella reviews (2020). JBI Manual for Evidence Synthesis.

[12] Hensher M, Cooper P, Dona S, Angeles M, Nguyen D, Heynsbergh N, Chatterton ML, Peeters A (2021). Scoping review: development and assessment of evaluation frameworks of mobile health apps for recommendations to consumers. J Am Med Inform Assoc.

[13] Nouri R, Kalhori SRN, Ghazisaeedi M, Marchand G, Yasini M (2018). Criteria for assessing the quality of mHealth apps: a systematic review. J Am Med Inform Assoc.

[14] Moshi MR, Tooher R, Merlin T (2018). Suitability of current evaluation frameworks for use in the health technology assessment of mobile medical applications: a systematic review. Int J Technol Assess Health Care.

[15] Lagan S, Sandler L, Torous J (2021). Evaluating evaluation frameworks: a scoping review of frameworks for assessing health apps. BMJ Open.

[16] Woulfe F, Fadahunsi KP, Smith S, Chirambo GB, Larsson E, Henn P, Mawkin M, O' Donoghue J (2021). Identification and evaluation of methodologies to assess the quality of mobile health apps in high-, low-, and middle-income countries: rapid review. JMIR Mhealth Uhealth.

[17] Velasco M, Perleth M, Drummond M, Gürtner F, Jørgensen T, Jovell A, Malone J, Rüther A, Wild C (2002). Best practice in undertaking and reporting health technology assessments. Working group 4 report. Int J Technol Assess Health Care.

[18] Merlin T, Tamblyn D, Ellery B, INAHTA Quality Assurance Group (2014). What's in a name? Developing definitions for common health technology assessment product types of the International Network of Agencies for Health Technology Assessment (INAHTA). Int J Technol Assess Health Care.

[19] Mobile health index and navigation database, app evaluation resources. MINDapps.

[20] Benjumea J, Ropero J, Rivera-Romero O, Dorronzoro-Zubiete E, Carrasco A (2020). Privacy assessment in mobile health apps: scoping review. JMIR Mhealth Uhealth.

[21] Nurgalieva L, O'Callaghan D, Doherty G (2020). Security and privacy of mHealth applications: a scoping review. IEEE Access.

[22] Muro-Culebras A, Escriche-Escuder A, Martin-Martin J, Roldán-Jiménez C, De-Torres I, Ruiz-Muñoz M, Gonzalez-Sanchez M, Mayoral-Cleries F, Biró A, Tang W, Nikolova B, Salvatore A, Cuesta-Vargas AI (2021). Tools for evaluating the content, efficacy, and usability of mobile health apps according to the consensus-based standards for the selection of health measurement instruments: systematic review. JMIR Mhealth Uhealth.

[23] Grundy Q (2022). A review of the quality and impact of mobile health apps. Annu Rev Public Health.

[24] Akbar S, Coiera E, Magrabi F (2020). Safety concerns with consumer-facing mobile health applications and their consequences: a scoping review. J Am Med Inform Assoc.

[25] Carmi L, Zohar M, Riva GM (2023). The European General Data Protection Regulation (GDPR) in mHealth: theoretical and practical aspects for practitioners' use. Med Sci Law.

[26] Galvin HK, DeMuro PR (2020). Developments in privacy and data ownership in mobile health technologies, 2016-2019. Yearb Med Inform.

[27] Azad-Khaneghah P, Neubauer N, Miguel Cruz A, Liu L (2021). Mobile health app usability and quality rating scales: a systematic review. Disabil Rehabil Assist Technol.

[28] General Data Protection Regulation.

[29] Marelli L, Lievevrouw E, van Hoyweghen I (2020). Fit for purpose? The GDPR and the governance of European digital health. Policy Studies.

[30] Hajesmaeel-Gohari S, Khordastan F, Fatehi F, Samzadeh H, Bahaadinbeigy K (2022). The most used questionnaires for evaluating satisfaction, usability, acceptance, and quality outcomes of mobile health. BMC Med Inform Decis Mak.

[31] Henson P, David G, Albright K, Torous J (2019). Deriving a practical framework for the evaluation of health apps. Lancet Digit Health.

[32] Maramba I, Chatterjee A, Newman C (2019). Methods of usability testing in the development of eHealth applications: a scoping review. Int J Med Inform.

[33] Zhou L, Bao J, Setiawan IMA, Saptono A, Parmanto B (2019). The mHealth App Usability Questionnaire (MAUQ): development and validation study. JMIR Mhealth Uhealth.

[34] Baumel A, Faber K, Mathur N, Kane JM, Muench F (2017). Enlight: a comprehensive quality and therapeutic potential evaluation tool for mobile and web-based ehealth interventions. J Med Internet Res.

