# Correction: Quality Assessment of Digital Health Apps: Umbrella Review

**DOI:** 10.2196/67849

**Published:** 2024-10-28

**Authors:** Maciej Marek Zych, Raymond Bond, Maurice Mulvenna, Jorge Martinez Carracedo, Lu Bai, Simon Leigh

**Affiliations:** 1 School of Computing Ulster University Belfast United Kingdom; 2 School of Electronics, Electrical Engineering and Computer Science Queen's University Belfast Belfast United Kingdom; 3 ORCHA Daresbury United Kingdom

In “Quality Assessment of Digital Health Apps: Umbrella Review” (J Med Internet Res 2024;26:e58616) one error has been noted.

Multimedia Appendix 1 presents the PRISMA checklist.

has been revised to:

Multimedia Appendix 1 presents the PRIOR checklist.

The correction will appear in the online version of the paper on the JMIR Publications website on October 28, 2024 together with the publication of this correction notice. Because this was made after submission to PubMed, PubMed Central, and other full-text repositories, the corrected article has also been resubmitted to those repositories.

