# Supplementary material for: Quality Assessment of Digital Health Apps: Umbrella Review
Source: J Med Internet Res. 2024 Oct 10;26:e58616. doi: 10.2196/58616 (PMC11502990; doi:10.2196/58616)
Supplement: Multimedia Appendix 2 [file jmir_v26i1e58616_app2.docx]

**Multimedia Appendix 2**

| **Database** | **Search query** | **Filters applied** | **Articles found** |
| --- | --- | --- | --- |
| IEEE Xplore | (("All Metadata":mhealth) OR ("All Metadata":ehealth) OR ("All Metadata":m-health) OR ("All Metadata":e-health) OR ("All Metadata":mobile health) OR ("All Metadata":electronic health) OR ("All Metadata":health app*) OR ("All Metadata":medical health app*) OR ("All Metadata":digital health app*) ("All Metadata":digital health product) OR ("All Metadata":digital health intervention) OR ("All Metadata":digital health technolog*) OR ("All Metadata":digital health solution)) AND (("All Metadata":assessment) OR ("All Metadata":assurance) OR ("All Metadata":audit)OR ("All Metadata":evaluation) OR ("All Metadata":framework)) AND (("All Metadata":review) OR ("All Metadata":assessment)) | journals, 2018-2023 | 1,581 |
| Scopus | ( ( mhealth OR ehealth OR m-health OR e-health OR "mobile health" OR "electronic health" OR "health app*" OR "medical health app*" OR "digital health app*" OR "digital health product*" OR "digital health intervention*" OR "digital health technolog*" OR "digital health solution*" ) AND ( assurance* OR assessment* OR evaluation* OR audit* OR framework* ) AND ( review* OR assessment* ) ) | Limited to review, Limited to English, Limited to Medicine, Limited to Computer Science, Year: 2018-2023 | 2,593 |
| PubMed | ((mhealth[Title/Abstract] OR ehealth[Title/Abstract] OR m-health[Title/Abstract] OR e-health[Title/Abstract] OR "mobile health"[Title/Abstract] OR "electronic health"[Title/Abstract] OR "health app*"[Title/Abstract] OR "medical health app*"[Title/Abstract] OR "digital health app*"[Title/Abstract] OR "digital health product*"[Title/Abstract] OR "digital health intervention*"[Title/Abstract] OR "digital health technolog*"[Title/Abstract] OR "digital health solution*"[Title/Abstract]) AND ( assurance*[Title/Abstract] OR assessment*[Title/Abstract] OR evaluation*[Title/Abstract] OR audit*[Title/Abstract] OR framework*[Title/Abstract]) AND (review*[Title/Abstract] OR assessment*[Title/Abstract])) | Full text, Review, Systematic Review, English, Exclude preprints, Humans, from 2018 - 2023. | 1,555 |
| ACM digital library | [[All: mhealth] OR [All: ehealth] OR [All: m-health] OR [All: e-health] OR [All: "mobile health"] OR [All: "electronic health"] OR [All: "health app*"] OR [All: "medical health app*"] OR [All: "digital health app*"] OR [All: "digital health product*"] OR [All: "digital health intervention*"] OR [All: "digital health technolog*"] OR [All: "digital health solution*"]] AND [[All: assurance*] OR [All: assessment*] OR [All: evaluation*] OR [All: audit*] OR [All: framework*]] AND [[All: review*] OR [All: assessment*]] AND [E-Publication Date: (01/01/2018 TO 31/12/2023)] | Review article, year 2018 - 2023 | 66 |
