# Supplementary material for: Quality Assessment of Digital Health Apps: Umbrella Review
Source: J Med Internet Res. 2024 Oct 10;26:e58616. doi: 10.2196/58616 (PMC11502990; doi:10.2196/58616)
Supplement: Multimedia Appendix 3 [file jmir_v26i1e58616_app3.docx]

**Multimedia Appendix 3: JBI critical appraisal of systematic reviews.**

| **Questions^1^** | **Systematic reviews** | | | |
| --- | --- | --- | --- | --- |
|  | (Muro-Culebras et al., 2021) | (Nouri et al., 2018) | (Moshi et al., 2018) | (Azad-Khaneghah et al., 2021) |
| 1. Is the review question clearly and explicitly stated? | Yes | Yes | Yes | Yes |
| 2. Were the inclusion criteria appropriate for the review question? | Yes | Yes | Yes | Yes |
| 3. Was the search strategy appropriate? | Yes | Yes | Yes | Yes |
| 4. Were the sources and resources used for the study adequate? | Yes | Yes | Yes | Yes |
| 5. Were the criteria for appraising studies appropriate? | Yes | NA | NA | Yes |
| 6. Was critical appraisal conducted by two or more reviewers independently? | Yes | NA | NA | Yes |
| 7. Were there methods to minimize errors in data extraction? | Yes | Yes | Yes | Yes |
| 8. Were the methods used to combine studies appropriate? | Yes | Yes | Yes | Yes |
| 9. Was the likelihood of publication bias assessed? | NA | NA | NA | Yes |
| 10. Were recommendations for policy and/or practice supported by the reported data? | Yes | Yes | Yes | Yes |
| 11. Were the specific directives for new research appropriate? | Yes | Yes | Yes | Yes |
| Overall appraisal^2^ | Include | Include | Include | Include |

^1^Possible responses: yes/no/unclear/not applicable.  ^2^ Possible responses: include/exclude/seek further information.

**References**

Azad-Khaneghah P, Neubauer N, Miguel Cruz A, Liu L. Mobile health app usability and quality rating scales: a systematic review. Disabil Rehabil Assist Technol 2021;16:712–21. <https://doi.org/10.1080/17483107.2019.1701103>.

Moshi MR, Tooher R, Merlin T. Suitability of current evaluation frameworks for use in the health technology assessment of mobile medical applications: a systematic review. Int J Technol Assess Health Care 2018;34:464–75. https://doi.org/10.1017/S026646231800051X.

Muro-Culebras A, Escriche-Escuder A, Martin-Martin J, Roldán-Jiménez C, De-Torres I, uiz-Muñoz M, et al. Tools for evaluating the content, efficacy, and usability of mobile health apps according to the consensus-based standards for the selection of health measurement instruments: systematic review. JMIR Mhealth Uhealth 2021;9:e15433. <https://doi.org/10.2196/15433>.

Nouri R, Kalhori SRN, Ghazisaeedi M, Marchand G, Yasini M. Criteria for assessing the quality of mHealth apps: a systematic review. J Am Med Inform Assoc 2018;25:1089–98. https://doi.org/10.1093/JAMIA/OCY050.
