# Supplementary material for: Quality Assessment of Digital Health Apps: Umbrella Review
Source: J Med Internet Res. 2024 Oct 10;26:e58616. doi: 10.2196/58616 (PMC11502990; doi:10.2196/58616)
Supplement: Multimedia Appendix 4 [file jmir_v26i1e58616_app4.docx]

**Multimedia Appendix 4: Characteristics of included reviews.**

| **Authors/Year** | **Objective** | **Total Sample size (may include duplicates)** | **Number of sources searched** | **Date (year) range of searched/**  **included studies** | **Number of studies included** | **Methods of analysis** | **Key findings** |
| --- | --- | --- | --- | --- | --- | --- | --- |
| (Muro-Culebras et al., 2021) | To psychometrically analyse (using COSMIN guideline) tools used to measure digital health app quality. | 3372 initial articles | 3 (PubMed, Embase databases and ‘other sources’) | Searched from February 1, 2019, to December 31, 2019 | 10 articles | Systematic review | The most validated tools were: The Health Information Technology Usability Evaluation Scale and the Measurement Scales for Perceived Usefulness and Perceived Ease of Use. The Mobile App Rating Scale had a moderate number of validated psychometric properties |
| (Nouri et al., 2018) | To review existing assessment of methods/tools for mHealth apps and provide classification of criteria. | 1057 initial articles | 4 (Web of Science, Scopus, Embase, Medline databases and ‘other sources’) | Searched from January 1, 2008, to December 22, 2016 | 23 articles | Systematic review | 7 main groups of criteria: design, information/content, usability, functionality, ethical issues, security and privacy, and User-perceived value. Each of these classes was divided into various sub-classes. In total, we identified 37 sub-classes of criteria. |
| (Moshi et al., 2018) | To review assessment frameworks for mobile medical applications and check for suitability for use in health technology assessment. | 12690 initial articles | 7 (PubMed (Medline), Embase, CINAHL, PsychInfo, The Cochrane Library, Compendex, Business Source Complete databases and ‘other sources’) | Searched from January 1, 2008, to October 31, 2016 | 46 articles | Systematic review | None of the evaluation frameworks could be used, unaltered, to guide the health technology assessment (HTA) of mobile medical applications (MMAs). To be used in HTA improvement regarding relevant comparators, assessments of harms and ongoing effect of software updates on the safety, effectiveness of MMAs, ethical issues, such as data privacy, and technology specific characteristics are needed. |
| (Azad-Khaneghah et al., 2021) | To review rating scales of tools used to measure quality and usability. As well as to compare their purpose, content, and target users. | 4402 initial articles | 5 (Medline, CINAHL, PsycINFO, IEEE Explore databases, and ‘other sources’) | Searched from July 31, 2018, to January 1, 2000 | 87 articles | Systematic review | Mobile application rating scale (MARS) is the most popular assessment tool for digital health apps quality. The review identified 25 app quality rating scales that included assessment criteria and 23 usability assessment scales. Current app quality rating scales are diverse in terms of criteria that they rate as measures of app quality. Mental Health Commission of Canada (MHCC) framework may improve rating scales for quality assessments. |
| (Nurgalieva et al., 2020) | To overview and synthesise research studies that assess security and privacy of mHealth apps. To review research-based design recommendations. | 83 articles | 3 (Scopus, PubMed, and ProQuest databases) | Searched from April 2016 to August 2019 | 83 articles | Scoping review | Evaluation of self-declared data from app developers was the most common privacy assessment technique. Few studies mentioned and fewer focused on compliance with existing regulations in their evaluations and design recommendations. |
| (Benjumea et al., 2020) | To understand how privacy of mHealth apps is assessed | 710 initial articles | 4 (Scopus, PubMed, IEEE Xplore, ACM Digital Library databases) | Searched since 2009 (included 2014 -onwards) | 24 articles | Scoping review | For user interfaces and privacy policies the criteria were very heterogeneous and less objective specifically for privacy policies, which can lead to irreproducible results. The study found it is often unclear what types of personal information is collected by the apps |
| (Lagan et al., 2021) | To review assessment frameworks published since 2018. To demonstrate comprehensiveness of proposed model by comparing it to existing frameworks. | 3886 initial articles | 3 (PubMed, EMBASE, PsychInfo databases and ‘other sources’) | Searched from January 2018 to October 2020 | 34 articles (from search in this study) and 45 (frameworks included from different search) | Scoping review | It is unclear whether any framework adequately predicts engagement. The study highlighted flexibility and comprehensiveness of the MIND framework. Despite the different disease conditions frameworks addressed, there was substantial overlap among the frameworks, especially around clinical foundation and privacy and security questions. The study results suggest while new app evaluation frameworks are developed, there is an appearing standard of common questions asked across all. |
| (Hensher et al., 2021) | To review assessment frameworks for their items. To analyse the scoring and assessment methods | 2143 initial articles | 7 (Medline complete, CINAHL complete, PubMed, Embase, Scopus, Google, Google Scholar databases and ‘other sources’) | Searched from January 2018 to April 2020 | 97 articles | Scoping review | The review identified 10 domains for digital health app evaluation: Clarity of purpose of the app, Developer credibility, Content/information validity, User experience, User engagement/adherence and social support, Interoperability, Value, Technical features and support, Privacy/security/ethical  /legal, and Accessibility |
| (Akbar et al., 2020) | To summarize research about safety of mHealth apps and their consequences. | 2388 initial articles | 4 (PubMed, WoS, Scopus, and Cochrane) | Searched from January 2013 to May 2019 | 74 articles | Scoping review | Safety concerns related to quality of information for digital health apps can be grouped into 5 categories: Incorrect information, incomplete information, variation in content, incorrect output, and inappropriate response to consumer needs. Safety concern related to software functionality can be grouped into 5 categories: Gaps in features, lack of validation for user input, delayed processing, response to health dangers, and faulty alarms. |
| (Maramba et al., 2019) | To summarise currents methods used in the assessment of usability of eHealth apps. | 1420 initial articles | 4 (ACM Digital Library, CINAHL, IEEE Xplore, and Medline / PubMed) | Included From April 2014 to October 2017 | 133 articles | Scoping review | 6 usability testing methods were identified in the reviewed articles: Two quantitative methods: Questionnaires, and task completion.  Three qualitative methods: “Think-Aloud” protocol, interviews and focus groups, and heuristic testing. |
| (Grundy, 2022) | To provide overview of mobile health apps and describe key stakeholders, in terms of how they are used, developed and regulated. | NA | NA | NA | NA | Narrative review | Recent surveys suggest that the development of new health apps is outpacing demand. Users seem to desire greater integration with their existing care system. App stores remain de facto regulators of digital health apps. Majority of digital health apps are not evidence based or do not follow public health guidelines. |
| (Galvin & DeMuro, 2020) | To review the literature regarding privacy protections and data ownership in mobile health technologies. | Not stated | 4 (PubMed, Google Scholar, and Web of Science, and ‘other sources’) | Searched from January 1, 2016 and June 1, 2019 | NA | Narrative review | The review states that data ownership regarding mobile health is a complicate manner due to many stakeholders. General Data Protection Regulation (GDPR) is increasing influencing the international regulatory framework. The review states that legislation is unable to keep up with the rapidly-advancing technology and consumer education and self-advocacy is limited. The review also states that fiduciary has a legal obligation to act in the best interest of a client. The review argues that entities which hold personal health data be therefore considered “health care information fiduciaries.” |
| (Hajesmaeel-Gohari et al., 2022) | To review and introduce the frequently used questionnaires for evaluating mobile health services (specifically: satisfaction, usability, acceptance, and quality). | 1028 initial articles | 1 (PubMed) | Searched on 18 April 2021 without date restriction | 247 articles | Narrative review | The review found that questionnaires with fewer items and higher reliability have been used more frequently by researchers. |
| (Carmi et al., 2022) | To examine privacy and data protection mechanisms related to mobile health and how to interpret regulatory requirements of General Data Protection Regulation (GDPR). | NA | NA | NA | NA | Narrative review | Interpretation of General Data Protection Regulation (GDPR) for mobile health. |
| (Woulfe et al., 2021) | To review current methodologies to assess the quality of mHealth apps and examine their applicability to low- medium- income countries. | 841 initial articles | 4 (PubMed, Embase, Scopus and WoS databases) | Searched 2005-2020 | 52 articles | Rapid review | An all-encompassing way for evaluating the quality of mHealth does not exist. Mobile application rating scale (MARS) fails to address some important key aspects of quality, most notably security and privacy. Enlight suite of assessments provides a more thorough assessment of quality. |

**References**

Akbar S, Coiera, E, Magrabi F. Safety concerns with consumer-facing mobile health applications and their consequences: a scoping review. Journal of the American Medical Informatics Association 2020;27:330–40. https://doi.org/10.1093/JAMIA/OCZ175.

Azad-Khaneghah P, Neubauer N, Miguel Cruz A, Liu L. Mobile health app usability and quality rating scales: a systematic review. Disabil Rehabil Assist Technol 2021;16:712–21. <https://doi.org/10.1080/17483107.2019.1701103>.

Benjumea J, Ropero J, Rivera-Romero O, Dorronzoro-Zubiete E, Carrasco A. Privacy Assessment in Mobile Health Apps: Scoping Review. JMIR Mhealth Uhealth 2020;8:e18868. https://doi.org/10.2196/18868.

Carmi L, Zohar M, Riva GM. The European General Data Protection Regulation (GDPR) in mHealth: Theoretical and practical aspects for practitioners’ use. Https://DoiOrg/101177/00258024221118411 2022;63:61–8. https://doi.org/10.1177/00258024221118411.

Galvin HK, DeMuro PR. Developments in Privacy and Data Ownership in Mobile Health Technologies, 2016-2019. Yearb Med Inform 2020;29:32. https://doi.org/10.1055/S-0040-1701987

Grundy Q. A Review of the Quality and Impact of Mobile Health Apps. Https://DoiOrg/101146/Annurev-Publhealth-052020-103738 2022;43:117–34. https://doi.org/10.1146/ANNUREV-PUBLHEALTH-052020-103738.

Hajesmaeel-Gohari S, Khordastan F, Fatehi F, Samzadeh H, Bahaadinbeigy K. The most used questionnaires for evaluating satisfaction, usability, acceptance, and quality outcomes of mobile health. BMC Med Inform Decis Mak 2022;22:1–9. https://doi.org/10.1186/S12911-022-01764-2/TABLES/2.

Hensher M, Cooper P, Dona SWA, Angeles MR, Nguyen D, Heynsbergh N, et al. Scoping review: Development and assessment of evaluation frameworks of mobile health apps for recommendations to consumers. Journal of the American Medical Informatics Association 2021;28:1318–29. https://doi.org/10.1093/JAMIA/OCAB041.

Lagan S, Sandler L, Torous J. Evaluating evaluation frameworks: a scoping review of frameworks for assessing health apps. BMJ Open 2021;11:e047001. https://doi.org/10.1136/BMJOPEN-2020-047001.

Maramba I, Chatterjee A, Newman C. Methods of usability testing in the development of eHealth applications: A scoping review. Int J Med Inform 2019;126:95–104. https://doi.org/10.1016/J.IJMEDINF.2019.03.018.

Moshi MR, Tooher R, Merlin T. Suitability of current evaluation frameworks for use in the health technology assessment of mobile medical applications: a systematic review. Int J Technol Assess Health Care 2018;34:464–75. https://doi.org/10.1017/S026646231800051X.

Muro-Culebras A, Escriche-Escuder A, Martin-Martin J, Roldán-Jiménez C, De-Torres I, uiz-Muñoz M, et al. Tools for evaluating the content, efficacy, and usability of mobile health apps according to the consensus-based standards for the selection of health measurement instruments: systematic review. JMIR Mhealth Uhealth 2021;9:e15433. <https://doi.org/10.2196/15433>.

Nouri R, Kalhori SRN, Ghazisaeedi M, Marchand G, Yasini M. Criteria for assessing the quality of mHealth apps: a systematic review. J Am Med Inform Assoc 2018;25:1089–98. https://doi.org/10.1093/JAMIA/OCY050.

Nurgalieva L, O’Callaghan D, Doherty G. Security and Privacy of mHealth Applications: A Scoping Review. IEEE Access 2020;8:104247–68. https://doi.org/10.1109/ACCESS.2020.2999934.

Woulfe F, Fadahunsi KP, Smith S, Chirambo GB, Larsson E, Henn P, et al. Identification and Evaluation of Methodologies to Assess the Quality of Mobile Health Apps in High-, Low-, and Middle-Income Countries: Rapid Review. JMIR Mhealth Uhealth 2021;9(10):E28384. https://MhealthJmirOrg/2021/10/E28384 2021;9:e28384. https://doi.org/10.2196/28384.
