# Supplementary material for: Quality Assessment of Digital Health Apps: Umbrella Review
Source: J Med Internet Res. 2024 Oct 10;26:e58616. doi: 10.2196/58616 (PMC11502990; doi:10.2196/58616)
Supplement: Multimedia Appendix 5 [file jmir_v26i1e58616_app5.docx]

**Multimedia Appendix 5**

|  | **Criteria** | | | | | | |  |
| --- | --- | --- | --- | --- | --- | --- | --- | --- |
| **Article reference** | Data privacy or security | Clinical assurance, credibility of information, or evidence | User experience, value, efficacy/effectiveness, or engagement | Safety | Features or functionality | Cost | Ethical or legal issues | **Total** |
| (Muro-Culebras et al., 2021) | ✓ | ✓ | ✓ | ✓ | X | X | X | 4 |
| (Nouri et al., 2018) | ✓ | ✓ | ✓ | ✓ | ✓ | X | ✓ | 6 |
| (Moshi et al., 2018) | ✓ | ✓ | ✓ | ✓ | ✓ | ✓ | ✓ | 7 |
| (Azad-Khaneghah et al., 2021) | ✓ | ✓ | ✓ | X | ✓ | X | X | 4 |
| (Nurgalieva et al., 2020) | ✓ | X | X | ✓ | X | X | ✓ | 3 |
| (Benjumea et al., 2020) | ✓ | X | X | ✓ | X | X | ✓ | 3 |
| (Lagan et al., 2021) | ✓ | ✓ | ✓ | X | ✓ | X | X | 4 |
| (Hensher et al., 2021) | ✓ | ✓ | ✓ | ✓ | ✓ | X | ✓ | 6 |
| (Akbar et al., 2020) | ✓ | ✓ | ✓ | ✓ | X | X | ✓ | 5 |
| (Maramba et al., 2019) | X | X | ✓ | X | X | X | X | 1 |
| (Grundy, 2022) | ✓ | ✓ | ✓ | ✓ | X | X | ✓ | 5 |
| (Galvin & DeMuro, 2020) | ✓ | ✓ | X | X | X | X | ✓ | 3 |
| (Hajesmaeel-Gohari et al., 2022) | X | ✓ | ✓ | X | X | X | X | 2 |
| (Carmi et al., 2022) | ✓ | X | X | X | X | X | ✓ | 2 |
| (Woulfe et al., 2021) | ✓ | ✓ | ✓ | ✓ | ✓ | X | ✓ | 6 |
| **Total** | 13 | 11 | 11 | 9 | 6 | 1 | 10 |  |

**References**

Akbar S, Coiera, E, Magrabi F. Safety concerns with consumer-facing mobile health applications and their consequences: a scoping review. Journal of the American Medical Informatics Association 2020;27:330–40. https://doi.org/10.1093/JAMIA/OCZ175.

Azad-Khaneghah P, Neubauer N, Miguel Cruz A, Liu L. Mobile health app usability and quality rating scales: a systematic review. Disabil Rehabil Assist Technol 2021;16:712–21. <https://doi.org/10.1080/17483107.2019.1701103>.

Benjumea J, Ropero J, Rivera-Romero O, Dorronzoro-Zubiete E, Carrasco A. Privacy Assessment in Mobile Health Apps: Scoping Review. JMIR Mhealth Uhealth 2020;8:e18868. https://doi.org/10.2196/18868.

Carmi L, Zohar M, Riva GM. The European General Data Protection Regulation (GDPR) in mHealth: Theoretical and practical aspects for practitioners’ use. Https://DoiOrg/101177/00258024221118411 2022;63:61–8. https://doi.org/10.1177/00258024221118411.

Galvin HK, DeMuro PR. Developments in Privacy and Data Ownership in Mobile Health Technologies, 2016-2019. Yearb Med Inform 2020;29:32. https://doi.org/10.1055/S-0040-1701987

Grundy Q. A Review of the Quality and Impact of Mobile Health Apps. Https://DoiOrg/101146/Annurev-Publhealth-052020-103738 2022;43:117–34. https://doi.org/10.1146/ANNUREV-PUBLHEALTH-052020-103738.

Hajesmaeel-Gohari S, Khordastan F, Fatehi F, Samzadeh H, Bahaadinbeigy K. The most used questionnaires for evaluating satisfaction, usability, acceptance, and quality outcomes of mobile health. BMC Med Inform Decis Mak 2022;22:1–9. https://doi.org/10.1186/S12911-022-01764-2/TABLES/2.

Hensher M, Cooper P, Dona SWA, Angeles MR, Nguyen D, Heynsbergh N, et al. Scoping review: Development and assessment of evaluation frameworks of mobile health apps for recommendations to consumers. Journal of the American Medical Informatics Association 2021;28:1318–29. https://doi.org/10.1093/JAMIA/OCAB041.

Lagan S, Sandler L, Torous J. Evaluating evaluation frameworks: a scoping review of frameworks for assessing health apps. BMJ Open 2021;11:e047001. https://doi.org/10.1136/BMJOPEN-2020-047001.

Maramba I, Chatterjee A, Newman C. Methods of usability testing in the development of eHealth applications: A scoping review. Int J Med Inform 2019;126:95–104. https://doi.org/10.1016/J.IJMEDINF.2019.03.018.

Moshi MR, Tooher R, Merlin T. Suitability of current evaluation frameworks for use in the health technology assessment of mobile medical applications: a systematic review. Int J Technol Assess Health Care 2018;34:464–75. https://doi.org/10.1017/S026646231800051X.

Muro-Culebras A, Escriche-Escuder A, Martin-Martin J, Roldán-Jiménez C, De-Torres I, uiz-Muñoz M, et al. Tools for evaluating the content, efficacy, and usability of mobile health apps according to the consensus-based standards for the selection of health measurement instruments: systematic review. JMIR Mhealth Uhealth 2021;9:e15433. <https://doi.org/10.2196/15433>.

Nouri R, Kalhori SRN, Ghazisaeedi M, Marchand G, Yasini M. Criteria for assessing the quality of mHealth apps: a systematic review. J Am Med Inform Assoc 2018;25:1089–98. https://doi.org/10.1093/JAMIA/OCY050.

Nurgalieva L, O’Callaghan D, Doherty G. Security and Privacy of mHealth Applications: A Scoping Review. IEEE Access 2020;8:104247–68. https://doi.org/10.1109/ACCESS.2020.2999934.

Woulfe F, Fadahunsi KP, Smith S, Chirambo GB, Larsson E, Henn P, et al. Identification and Evaluation of Methodologies to Assess the Quality of Mobile Health Apps in High-, Low-, and Middle-Income Countries: Rapid Review. JMIR Mhealth Uhealth 2021;9(10):E28384. https://MhealthJmirOrg/2021/10/E28384 2021;9:e28384. https://doi.org/10.2196/28384.
